# Predictive nomogram for in-hospital mortality among older patients with intra-abdominal sepsis incorporating skeletal muscle mass

**DOI:** 10.1007/s40520-023-02544-2

**Published:** 2023-09-05

**Authors:** Qiujing Li, Na Shang, Tiecheng Yang, Qian Gao, Shubin Guo

**Affiliations:** 1 Department of Emergency Medicine, Beijing Shijitan Hospital, Capital Medical University, Beijing, China; 2 Department of Emergency Medicine, Capital Medical University of Rehabilitation Medicine, Beijing Bo’Ai Hospital, China Rehabilitation Research Center, Beijing, China; 3 Department of Emergency Medicine, Key Laboratory of Cardiopulmonary Cerebral Resuscitation, Beijing Chao-Yang Hospital, Capital Medical University, No. 8, South Road of Worker’s Stadium, BeijingChaoyang District, Beijing, 100020 China

**Keywords:** Intra-abdominal sepsis, Mortality, Sarcopenia, Older adults, Nomogram

## Abstract

**Background:**

Studies on prognostic factors for older patients with intra-abdominal sepsis are scarce, and the association between skeletal muscle mass and prognosis among such patients remains unclear.

**Aims:**

To develop a nomogram to predict in-hospital mortality among older patients with intra-abdominal sepsis.

**Methods:**

Older patients with intra-abdominal sepsis were prospectively recruited. Their demographics, clinical features, laboratory results, abdominal computed tomography-derived muscle mass, and in-hospital mortality were recorded. The predictors of mortality were selected via least absolute shrinkage and selection operator and multivariable logistic regression analyses, and a nomogram was developed. The nomogram was assessed and compared with Sequential Organ Failure Assessment score, Acute Physiology and Chronic Health Evaluation II score, and Simplified Acute Physiology Score II.

**Results:**

In total, 464 patients were included, of whom 104 (22.4%) died. Six independent risk factors (skeletal muscle index, cognitive impairment, frailty, heart rate, red blood cell distribution width, and blood urea nitrogen) were incorporated into the nomogram. The Hosmer–Lemeshow goodness-of-fit test and calibration plot revealed a good consistency between the predicted and observed probabilities. The area under the receiver operating characteristic curve was 0.875 (95% confidence interval = 0.838–0.912), which was significantly higher than those of commonly used scoring systems. The decision curve analysis indicated the nomogram had good predictive performance.

**Discussion:**

Our nomogram, which is predictive of in-hospital mortality among older patients with intra-abdominal sepsis, incorporates muscle mass, a factor that warrants consideration by clinicians. The model has a high prognostic ability and might be applied in clinical practice after external validation.

## Introduction

Sepsis, which is caused by a dysregulated host response to infection, is a life-threatening organ dysfunction with high morbidity and mortality worldwide [1]. Approximately 48.9 million sepsis cases and 11.0 million sepsis-related deaths were reported for 2017 [2]. More than 60% of sepsis cases occur in adults aged ≥ 65 years [3]. Intra-abdominal infection is the second most common cause of sepsis, with a mortality rate of 29.1–36.3% [4–6]. Older age is independently associated with mortality in patients with intra-abdominal infection and sepsis [5]; therefore, older patients with intra-abdominal sepsis warrant further investigation.

The identification of prognostic factors for sepsis is of great value for accurate stratification, medical decision-making, and even improving prognosis. Several risk factors for mortality among patients with intra-abdominal sepsis have been reported, including coagulation dysfunction, acute kidney injury, and sarcopenic obesity [7, 8]. However, to date, no studies on the prognostic factors in older patients with intra-abdominal sepsis have been performed, and no specific prediction model is available.

Abdominal computed tomography (CT) can provide diagnostic information regarding intra-abdominal sepsis and can be used to measure skeletal muscle mass [9]. Loss of muscle mass or sarcopenia was associated with a poor prognosis of patients with sepsis [10–12]. However, the association between muscle mass and prognosis among older patients with intra-abdominal sepsis remains unclear.

Therefore, in this study, we aimed to investigate prognostic factors among older patients with intra-abdominal sepsis and determine the association between muscle mass and mortality. Furthermore, we developed a prognostic model for such patients by using a nomogram and compared it with commonly used scoring systems, including the Sequential Organ Failure Assessment (SOFA) score, Acute Physiology and Chronic Health Evaluation (APACHE) II score, and Simplified Acute Physiology Score (SAPS) II.

## Methods

### Study design and participants

This prospective, observational cohort study was conducted at the emergency department (ED) of Beijing Chao-Yang Hospital, Capital Medical University, China from January 1, 2022 to November 30, 2022. The study protocol was approved by the Institutional Review Board of the Beijing Chao-Yang Hospital, Capital Medical University (approval no.: 2022-ke-430). Informed consent was obtained from each participant or their next of kin. During the study period, patients who tested negative for coronavirus disease 2019 (COVID-19) were admitted to our hospital, whereas those with COVID-19 were transferred to government-designated hospitals.

Older patients (≥ 65 years) with intra-abdominal sepsis who underwent abdominal CT within 72 h of ED admission were enrolled and followed up until the time of hospital discharge or death. Sepsis was defined according to the Sepsis-3 criteria, that is, a suspected or confirmed infection plus an acute increase in the SOFA score ≥ 2 points [1]. Biliary tract infection, pancreatic infection, intra-abdominal abscess, peritonitis caused by intestinal obstruction or perforation of the gastrointestinal tract, enteritis, colitis, appendicitis, and colon cancer were defined as intra-abdominal infections. The exclusion criteria were as follows: (a) patients who were discharged or transferred within 24 h of admission; (b) patients with neuromuscular disease or cachexia; (c) patients for whom CT images did not meet the quality checks; and (d) patients with a missing information rate of > 20%.

### Demographic and clinical information

The following clinical data were recorded within 24 h of admission to the ED. (a) Demographic data: age, sex, and body mass index (BMI). (b) Comprehensive geriatric assessment: presence of cognitive impairment, bedridden status, Barthel index, and frailty. Mini Mental State Examination (MMSE) was used for the assessment of cognitive impairment, patients with a score of 26 or less were defined as having cognitive impairment [13]. Frailty was assessed with Clinical frailty scale (CFS), which was obtained by evaluating patient pre-hospitalization mobility and function assessments [14]. CFS is a nine-point global frailty scale, ranging from 1 (very fit) to 9 (terminally ill), patients with a score of 5 or more were classified as frail [15]. (c) Comorbidities: hypertension, coronary heart disease, diabetes, solid and hematological malignancy, chronic kidney disease, chronic lung disease, chronic liver disease, and connective tissue disease. (d) Etiology of intra-abdominal sepsis: biliary tract infection, pancreatic infection, intra-abdominal abscess, peritonitis caused by intestinal obstruction or perforation of the gastrointestinal tract, enteritis, colitis, appendicitis, and colon cancer. (e) Vital signs: temperature, heart rate (HR), respiratory rate, mean arterial pressure, and oxygenation index (PaO_2_/FiO_2_). (f) The first clinical laboratory values after admission to the ED: complete blood cell count, liver and renal function, electrolyte concentrations, osmotic pressure, coagulation, D-dimer concentration, and procalcitonin (PCT) concentration. (g) Scoring systems for older patients with intra-abdominal sepsis: the SOFA score, APACHE II score, and SAPS II, which were calculated as the worst laboratory indicators within 24 h of admission.

### CT-based muscle mass measurement

CT images were retrieved from the institutional picture archiving and communication system and analyzed using the AW Volume Share 7 workstation (GE Medical Systems S.C.S). Its CT histogram software “X-Section” was used to manually delineate the region of interest (ROI) and automatically calculate the ROI area. The skeletal muscle area (SMA) at the midpoint of the third lumbar vertebra (L3) in the transverse CT image, which is reportedly significantly correlated with whole-body muscle mass [9], was measured using Hounsfield unit (HU) thresholds (−29 to + 150 HU) [16]. The skeletal muscle index (SMI) was calculated as the SMA (cm^2^) divided by the patient’s height squared (m^2^) [9]. All measurements were obtained by the same emergency physician who was blinded to the patients' prognoses and trained to measure muscle mass.

### Study outcome

The primary clinical outcome was all-cause in-hospital mortality. Clinical data were compared between survival and non-survival groups.

### Statistical analysis

Statistical analyses were conducted using IBM SPSS Statistics for Windows version 26.0 (IBM Corp., Armonk, NY, USA) and R software version 4.2.2 (R Foundation for Statistical Computing, Vienna, Austria). Continuous variables were described as the mean (standard deviation) or median (interquartile range [IQR]) and compared using Student’s t test or Mann–Whitney U test. Categorical variables were expressed as frequencies (percentages) and compared using the chi-square test. For variables with < 20% missing data, such data were imputed using the multiple imputation method in SPSS. The least absolute shrinkage and selection operator (LASSO) procedure was used to select prognostic variables via the “glmnet” R package. Selected variables were included in the multivariable logistic regression model, and a forward stepwise method was used to determine the independent risk factors. Based on the independent variables, we developed the mortality prediction model and constructed a nomogram by using the “rms” R package. We used a calibration plot and Hosmer–Lemeshow goodness-of-fit test to evaluate the degree of calibration of the nomogram. We used receiver operating characteristic (ROC) curves, the area under the ROC curve (AUC), category-free net reclassification improvement (NRI), and integrated discrimination improvement (IDI) to assess the discriminative ability of the nomogram and compare it with that of the SOFA score, SAPS II, and APACHE II score. Decision curve analysis (DCA) was used to assess the clinical utility of the model.

All tests were two-sided, and differences were considered significant when the *p* value was < 0.05.

## Results

### Study population

**Fig. 1:**
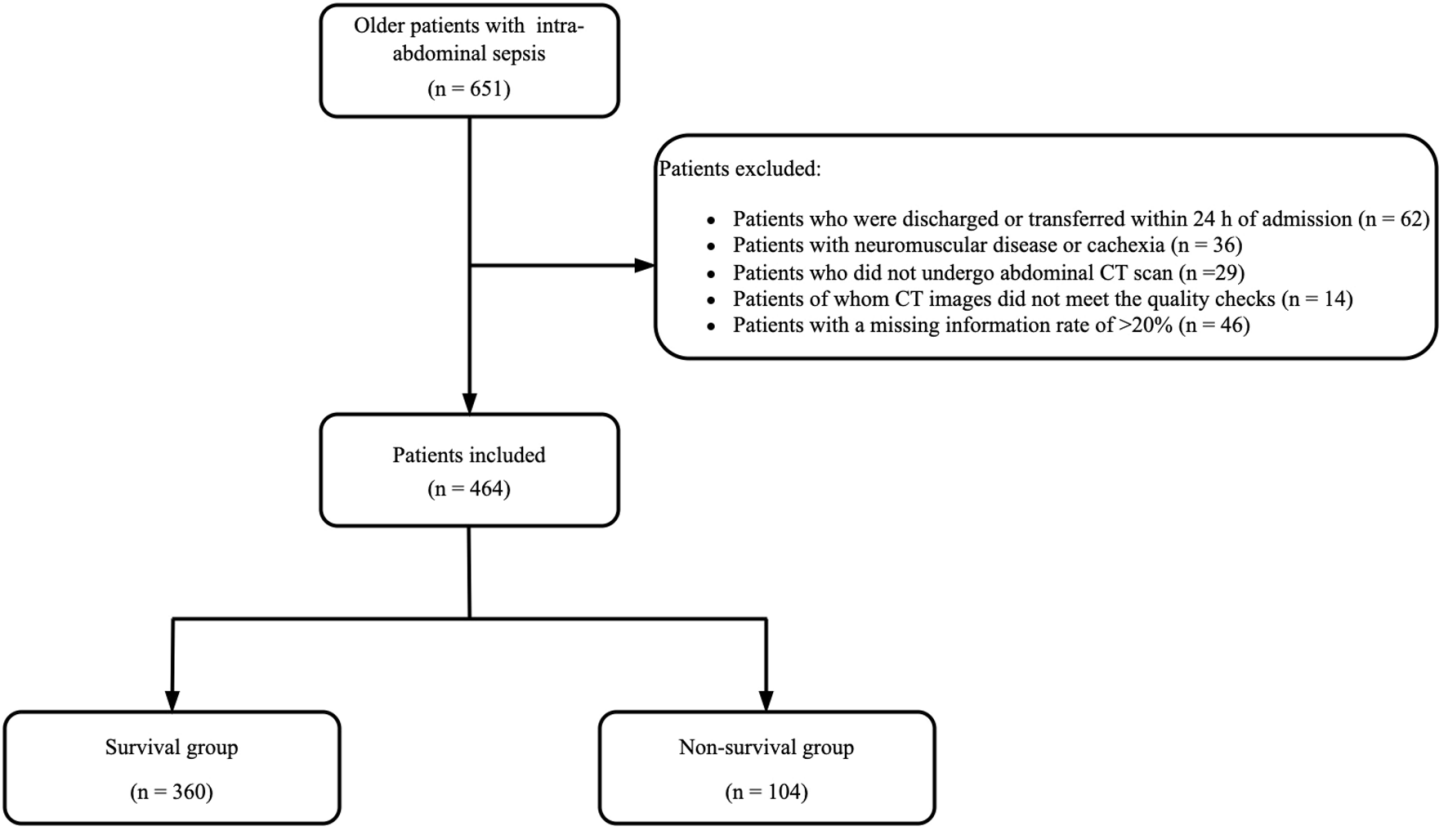
Flowchart of the study cohort

In total, 651 older patients were diagnosed with intra-abdominal sepsis. We excluded 62 patients who were discharged or transferred within 24 h, 36 with neuromuscular disease or cachexia, 29 without abdominal CT images, 14 for whom the CT images did not meet the quality checks, and 46 with a missing information rate of > 20%. Therefore, 464 patients were included and followed-up. Among them, the in-hospital mortality rate was 22.4% (104 patients died) (Fig. 1).

### Clinical characteristics

The demographic and clinical characteristics of the patients are listed in Table 1 . Their median age was 76 (IQR, 16) years, 222 (47.8%) were men, their median SMI was 34.17 (IQR, 11.44) cm^2^/m^2^, 143 (30.8%) had cognitive impairment, 240 (51.7%) were frail, and 128 (27.6%) were bedridden. Compared with the survival group, the non-survival group was older (median age, 75 vs. 82 years); had higher prevalence of cognitive impairment (20.8% vs. 65.4%), frailty (41.4% vs. 87.5%), bedriddenness (19.4% vs. 55.8%), and septic shock (24.2% vs. 56.7%); and had a higher Charlson Comorbidity Index (5 vs. 6), SOFA score (3 vs. 7), APACHE II score (9 vs. 18), and SAPS II (33 vs. 47, all *p* < 0.001). Meanwhile, non-survivors had a lower BMI (24.0 vs. 22.3 kg/m^2^), SMI (35.63 vs. 31.19 cm^2^/m^2^), and Barthel index (70 vs. 58, all *p* < 0.001).

**Table 1:** Demographic and clinical characteristics of survival and non-survival older patients with intra-abdominal sepsis

Variables	Total (*n* = 464)	survival group (*n* = 360)	non-survival group (*n* = 104)	*p* value
Age (years), median (IQR)	76 (17)	75 (17)	82 (13)	<0.001
Male, *n* (%)	222 (47.8)	170 (47.2)	52 (50.0)	0.617
BMI (kg/m^2^), mean (SD)	23.6 (3.7)	24.0 (3.6)	22.3 (3.8)	<0.001
SMI (cm^2^/m^2^), median (IQR)	34.17 (11.44)	35.63 (11.03)	31.19 (8.09)	<0.001
Comprehensive geriatric assessment				
Cognitive impairment, *n* (%)	143 (30.8)	75 (20.8)	68 (65.4)	<0.001
Frailty, *n* (%)	240 (51.7)	149 (41.4)	91 (87.5)	<0.001
Barthel index, median (IQR)	80 (80)	93 (55)	20 (64)	<0.001
Bedridden status, *n* (%)	128 (27.6)	70 (19.4)	58 (55.8)	<0.001
Comorbidities, *n* (%)				
Hypertension	213 (45.9)	150 (41.7)	63 (60.6)	0.001
Coronary heart disease	119 (25.6)	89 (24.7)	30 (28.8)	0.396
Diabetes	149 (32.1)	112 (31.1)	37 (35.6)	0.390
Solid and hematological malignancy	68 (14.7)	46 (12.8)	22 (21.2)	0.033
Chronic kidney disease	60 (12.9)	40 (11.1)	20 (19.2)	0.030
Chronic lung disease	33 (7.1)	23 (6.4)	10 (9.6)	0.259
Chronic liver disease	16 (3.4)	12 (3.3)	4 (3.8)	0.801
Connective tissue disease	13 (2.8)	10 (2.8)	3 (2.9)	0.954
Charlson comorbidity index, median (IQR)	5 (2)	5 (3)	6 (3)	<0.001
Etiology of intra-abdominal sepsis, *n* (%)				0.089
Biliary tract infection	183 (39.4)	142 (39.4)	41 (39.4)	
Pancreatic infection	86 (18.5)	68 (18.9)	18 (17.3)	
Intra-abdominal abscess	60 (12.9)	47 (13.1)	13 (12.5)	
Peritonitis caused by intestinal obstruction	62 (13.4)	42 (11.7)	20 (19.2)	
Peritonitis caused by perforation of gastrointestinal tract	32 (6.9)	23 (6.4)	9 (8.7)	
Enteritis, colitis, appendicitis and colon cancer	41 (8.8)	38 (10.6)	3 (2.9)	
Severe scoring systems, median (IQR)				
SOFA score	3 (4)	3 (3)	7 (4)	<0.001
APACHE II score	11 (11)	9 (9)	18 (11)	<0.001
SAPS II	36 (17)	33 (15)	47 (23)	<0.001
Septic shock, *n* (%)	146 (31.5)	87 (24.2)	59 (56.7)	<0.001
LOS, median (IQR)	11.0 (8.0)	12.0 (7.0)	8.5 (15.0)	0.123

*IQR* interquartile range, *BMI* body mass index, *SD* standard deviation, *SMI* skeletal muscle index, *LOS* length of stay, *SOFA score* Sequential Organ Failure Assessment score, *APACHE II score* Acute Physiology and Chronic Health Evaluation II score, *SAPS II* Simplified Acute Physiology Score II

### Candidate predictor selection

Fifty-five candidate variables, including age, sex, BMI, SMI, comprehensive geriatric assessment, comorbidities, septic shock, vital signs, complete blood cell count, liver and renal function, electrolyte concentrations, osmotic pressure, coagulation test results, D-dimer concentration, and PCT concentration, were included in the LASSO regression model. The following 11 predictors were associated with in-hospital mortality when the optimal λ value was 0.039: SMI, cognitive impairment, Barthel index, frailty, septic shock, HR, red blood cell distribution width (RDW), prealbumin concentration, blood urea nitrogen (BUN), K^+^, and international normalized ratio. Upon multivariable logistic regression analysis, six independent risk factors were identified: SMI (odds ratio [OR] = 0.955, 95% confidence interval [CI] = 0.919–0.992, *p* = 0.019), cognitive impairment (OR = 3.050, 95% CI = 1.669–5.572, *p* < 0.001), frailty (OR = 3.187, 95% CI = 1.533–6.625, *p* = 0.002), HR (OR = 1.018, 95% CI = 1.006–1.031, *p* = 0.003), RDW (OR = 1.279, 95% CI = 1.144–1.429, *p* < 0.001), and BUN (OR = 1.057, 95% CI = 1.032–1.082, *p* < 0.001) (Table 2).

**Table 2:** Univariable and multivariable logistic regression analysis of risk factors associated with in-hospital mortality among older patients with intra-abdominal sepsis

	Univariable analysis			Multivariable analysis*		
	OR	95%CI	*p* value	OR	95%CI	*p* value
SMI, cm^2^/m^2^	0.915	0.886–0.944	< 0.001	0.955	0.919–0.992	0.019
Cognitive impairment	7.178	4.453–11.570	< 0.001	3.050	1.669–5.572	< 0.001
Barthel index	0.974	0.968–0.981	< 0.001			
Frailty	9.913	5.344–18.387	< 0.001	3.187	1.533–6.625	0.002
Septic shock	4.114	2.605–6.497	< 0.001			
HR, bpm	1.030	1.019–1.041	< 0.001	1.018	1.006–1.031	0.003
RDW, %	1.338	1.223–1.464	< 0.001	1.279	1.144–1.429	< 0.001
Prealbumin, g/L	0.001	0.000–0.013	< 0.001			
BUN, mmol/L	1.076	1.052–1.100	< 0.001	1.057	1.032–1.082	< 0.001
K^+^, mmol/L	1.740	1.326–2.282	< 0.001			
INR	6.494	2.758–15.289	< 0.001			

*Adjusted for Barthel index, presence of septic shock, prealbumin, K, INR.

*SMI* skeletal muscle index, *HR* heart rate, *RDW* red blood cell distribution width, *BUN* blood urea nitrogen, *INR* international normalized ratio, *OR* odds ratio, *CI* confidential interval

### Nomogram development

**Fig. 2:**
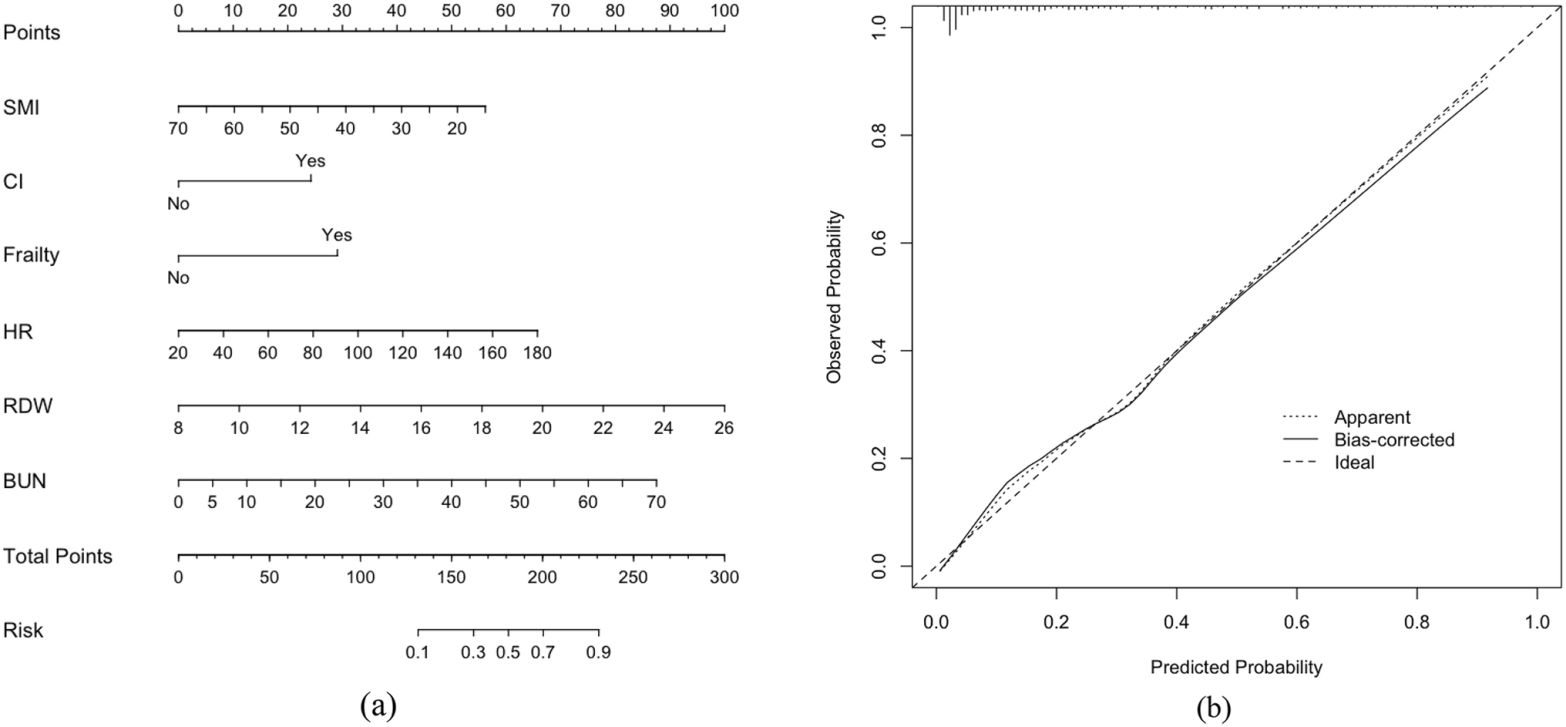
**(a)** Nomogram for predicting in-hospital mortality in older patients with intra-abdominal sepsis. **(b)** Calibration plot for the nomogram

A nomogram was developed based on the six independent prognostic factors for in-hospital mortality of older patients with intra-abdominal sepsis. The nomogram consisted of scoring each predictor on its corresponding scale and summing them to obtain the total points. On the bottom of the nomogram, the risk corresponding to the total points represented the probability of in-hospital mortality. According to the nomogram, patients with a lower SMI and higher HR, RDW, and BUN who presented with cognitive impairment and frailty had a higher total score and, thus, a higher risk of mortality (Fig. 2a).

### Nomogram calibration

The Hosmer–Lemeshow goodness-of-fit test indicated a good calibration of the nomogram model (*X*^2^ = 1.473, degrees of freedom = 2, *p* = 0.479). The calibration curve was nearly diagonal, indicating good agreement between the predicted and actual outcomes (Fig. 2b).

### Nomogram validation

**Fig. 3:**
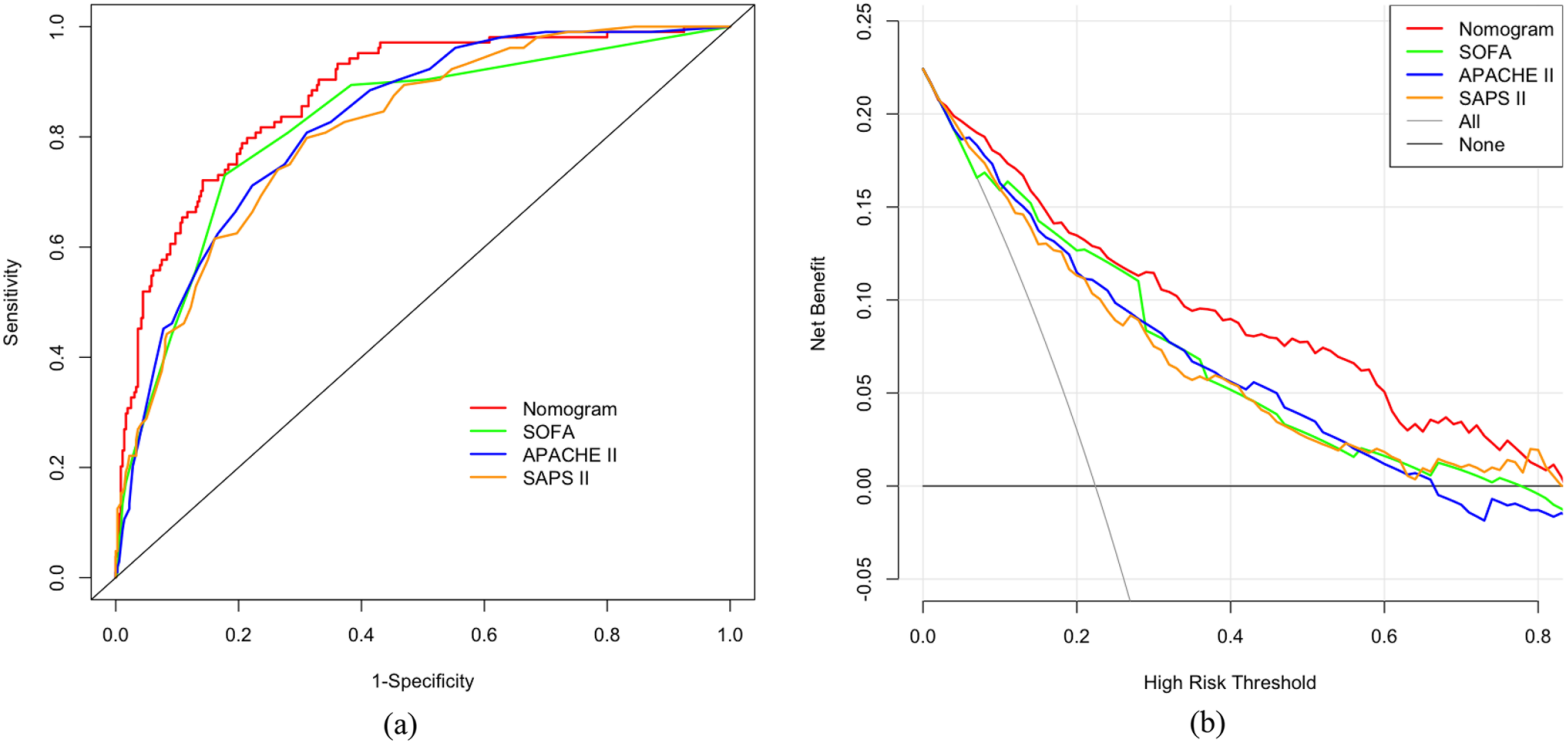
Comparison of the nomogram and commonly used scoring systems. **(a)** ROC curves; **(b)** decision curve analysis

We compared the discriminative capacities of the nomogram and commonly used scoring systems for the prediction of in-hospital mortality among older patients with intra-abdominal sepsis. The AUC for the nomogram was 0.875 (95% CI = 0.838–0.912), which was significantly higher than those for the SOFA score (0.822, 95% CI = 0.776–0.868, *p* = 0.048), APACHE II score (0.825, 95% CI = 0.783–0.867, *p* = 0.008), and SAPS II (0.812, 95% CI = 0.768–0.856, *p* = 0.001) (Table 3 ). The ROC curves and AUCs indicated that the nomogram had a significantly higher predictive ability for in-hospital mortality than the other tools (Fig. 3a). Moreover, in comparing the nomogram and commonly used scoring systems, both the NRI and IDI values were positive, and their corresponding *p* values were < 0.05 (Table 3 ), also indicating the nomogram’s superior predictive discrimination ability. In the DCA, the curve of the nomogram was higher than those of the other scoring systems, illustrating that clinical interventions guided by the nomogram would have higher net benefits (Fig. 3b).

**Table 3:** The AUC, NRI and IDI of different predictive models for in-hospital mortality among older patients with intra-abdominal sepsis

Predictive models	AUC	*p* value	NRI	*p* value	IDI	*p* value
Nomogram	0.875 (0.838–0.912)					
SOFA score	0.822 (0.776–0.868)	0.048	0.509 (0.296–0.722)	< 0.001	0.112 (0.052–0.171)	< 0.001
APACHE II score	0.825 (0.783–0.867)	0.008	0.612 (0.401–0.822)	< 0.001	0.121 (0.070–0.172)	< 0.001
SAPS II	0.812 (0.768–0.856)	0.001	0.686 (0.478–0.894)	< 0.001	0.130 (0.077–0.184)	< 0.001

*AUC* area under the receiver operating characteristic curve, *NRI* net reclassification improvement, *IDI* integrated discrimination improvement, *SOFA score* Sequential Organ Failure Assessment score, *APACHE II score* Acute Physiology and Chronic Health Evaluation II score, *SAPS II* Simplified Acute Physiology Score II

## Discussion

We conducted an observational, prospective study of 464 consecutive older patients with intra-abdominal sepsis to develop a novel prognostic nomogram for the prediction of in-hospital mortality. The nomogram was based on six risk factors: SMI, cognitive impairment, frailty, HR, RDW, and BUN. Notably, we measured abdominal CT-derived muscle mass (represented in the nomogram by the SMI) and demonstrated its association with in-hospital mortality. To our knowledge, this is the first study for which such prognostic factors were reported and a predictive model for mortality developed for older patients with intra-abdominal sepsis.

Several models were previously developed to predict mortality among patients with sepsis [17–19]. However, specific and simple tools for such prediction among older patients with intra-abdominal sepsis are lacking. Huang et al. constructed a nomogram for overall survival of patients with complicated intra-abdominal infections, which incorporated sex, acute kidney injury, acute gastrointestinal injury, rare bacterial infections, Charlson Comorbidity Index, and APACHE II score [20]. However, the Charlson Comorbidity Index and APACHE II scores are cumbersome to calculate. The variables in our nomogram are more readily obtained. Moreover, our nomogram outperformed the SOFA score, APACHE II score, and SAPS II in terms of predictive and discriminatory performance.

Given that most patients with intra-abdominal sepsis require abdominal CT, we considered abdominal CT-derived skeletal muscle mass as a potential predictor. Previous studies have revealed that loss of muscle mass or sarcopenia is associated with increased mortality among patients with sepsis [10–12, 21]. However, studies on the association of skeletal muscle mass and prognosis in older patients with intra-abdominal sepsis are scarce. Baggerman et al. discovered that muscle wasting-associated comorbidities, rather than sarcopenia, are associated with in-hospital mortality in critically ill patients with abdominal sepsis [22]. However, in our study, skeletal muscle mass, as a continuous variable, was an independent risk factor for in-hospital mortality. Population heterogeneity and different definitions of sarcopenia may account for these differences. Notably, as SMI values were different in various races, the scoring of the SMI values used in the nomogram may be different in the non-Asian population. Sarcopenia is commonly defined as a loss of skeletal muscle mass and decreased functional strength [23]. However, we did not consider muscle strength because patients in the ED, particularly those who are acutely critically ill, are not always able to complete physical function tests, such as walking speed, grip strength, or a short physical performance battery.

Frailty is characterized by a decline in function and increased vulnerability to stressors [14]. Previous studies have demonstrated a prevalence of frailty of 36.8–44.6% in the ED [24–26]. In our study, the prevalence of frailty was higher (51.7%). The reason for this difference is that our study population was older patients, and another reason is differences in disease severity among studies. Frailty in patients with suspected infection or sepsis is reportedly associated with mortality [27, 28]. Those results are consistent with our discovery that frailty is an independent risk factor for mortality in older patients with intra-abdominal sepsis.

The RDW is a simple parameter obtained with complete blood cell counts. A high RDW reflects anisocytosis, including impaired erythropoiesis and abnormal red blood cell survival [29]. Several studies have demonstrated that high RDW values are associated with poor prognoses in patients with various diseases [30–32]. In our study, RDW was also an independent risk factor for in-hospital mortality.

Additionally, we assessed the functional status and cognitive function of the participants using the Barthel index, cognitive impairment, and bedridden status. Among these, only cognitive impairment was an independent risk factor. Our nomogram also includes BUN and HR, both of which are readily available. These variables also formed a part of previous models [17, 33, 34].

Our study has some limitations. First, it was a single-center study, which might have contributed to selection bias. The missing data increased the risk of information bias, which might have limited the generalizability of the results. Second, certain risk factors, such as diffuse peritonitis, antimicrobial resistance, source control failure, and continuous renal replacement therapy, are reportedly associated with mortality in patients with intra-abdominal infections and sepsis [5, 7]. Although these risk factors were excluded from our study, we cannot exclude the presence of unadjusted confounders. Third, although the nomogram yielded superior discrimination and calibration to the SOFA score, APACHE II score, and SAPS II, no external validation was performed. This is an area for further research.

In summary, we developed a novel prognostic nomogram for in-hospital mortality among older patients with intra-abdominal sepsis. Compared with commonly used scoring systems, the nomogram yielded better discrimination and calibration, indicating that it is useful for early risk stratification and might be applied in clinical practice after external validation. Our results suggest that the assessment of skeletal muscle mass warrants more attention in clinical practice.

## Data Availability

The data generated and/or analyzed during this study is available from the corresponding author upon reasonable request.
